# Integrated proteomic and metabolomic analyses define the molecular architecture of acute mountain sickness

**DOI:** 10.3389/fgene.2026.1865110

**Published:** 2026-09-01

**Authors:** Wenjing Ding, Yifan Yang, Huaying Wei, Xinyu Hu, Haopeng Zhang, Ailifeila Aili, Xiaolan Chen, Qiqi Wang, Xinying Xue, Lei Pan

**Affiliations:** 1 Department of Respiratory and Critical Care, Emergency and Critical Care Medical Center, Beijing Shijitan Hospital, Capital Medical University, Beijing, China; 2 Department of Geriatrics, Beijing Friendship Hospital, Capital Medical University, Beijing, China; 3 Department of Respiratory and Critical Care, Xuanwu Hospital of Capital Medical University, National Clinical Research Center for Geriatric Diseases, Beijing, China; 4 Department of Respiratory and Critical Care, Shandong Second Medical University, Weifang, China

**Keywords:** acute mountain sickness, hypobaric hypoxia, machine learning, metabolomics, multi-omics, precision medicine, proteomics

## Abstract

**Background:**

Hypoxia-driven vascular, immune, and metabolic remodeling is a key biological process involved in cardiovascular diseases, cancer, and other complex systemic disorders. Acute mountain sickness (AMS) is an acute manifestation of hypobaric hypoxia, but its systemic molecular features remain incompletely defined.

**Methods:**

We performed integrated plasma proteomic and metabolomic profiling in 81 healthy Han Chinese male participants after rapid high-altitude exposure. Differential analysis, weighted gene co-expression network analysis (WGCNA), tissue-specific protein mapping, regulatory network reconstruction, machine learning, and druggability assessment were applied to characterize AMS-associated molecular alterations and identify candidate biomarkers and targets.

**Results:**

Multi-omics profiling identified 3,137 proteins and 4,104 metabolites and showed clear separation between AMS and non-AMS participants. AMS was characterized by coordinated thrombo-inflammatory activation, coagulation-related disturbance, and metabolic reprogramming, including suppression of oxidative phosphorylation-related signatures. WGCNA identified symptom associated proteomic and metabolomic modules linked to headache severity, oxygen saturation, and hemodynamic traits. Tissue-specific protein mapping revealed a liver-centered but multi-organ circulating proteomic architecture, suggesting hepatic secretory remodeling with additional neural and immune-system contributions. Regulatory network analysis highlighted NOTCH1 as a candidate upstream regulatory hub, whereas druggability analysis prioritized NOTCH1 and the antioxidant-related protein GSTA1 as translational candidates. An mRMR plus logistic regression classifier integrating 15 proteomic features and SpO2 achieved good discriminatory performance, with an AUC of 0.968 in the training cohort and 0.913 in the test cohort.

**Conclusion:**

This study defines a multi-layer molecular framework of human acute hypoxic stress, linking vascular regulation, inflammation, coagulation, metabolic remodeling, tissue origin, and biomarker prioritization. These findings provide mechanistic insight into AMS and support multi-omics-based biomarker discovery and target prioritization in hypoxia-associated systemic diseases.

## 1 Introduction

Rapid ascent to high altitude exposes the human body to hypobaric hypoxia, a major environmental stressor that disrupts oxygen homeostasis and triggers coordinated systemic responses. Among altitude-related illnesses, acute mountain sickness (AMS) is the most common clinical syndrome and is characterized by headache, dizziness, gastrointestinal discomfort, fatigue, and sleep disturbance (Roach et al., 1993; Luks et al., 2017; Gatterer et al., 2024). Although AMS is usually self-limiting, it reflects inadequate acclimatization to acute hypoxic stress and may precede more severe altitude-related disorders (Luks et al., 2017; Guo et al., 2022). Clinically, AMS assessment still relies predominantly on symptom-based tools such as the Lake Louise Score (LLS), which are practical for field use but provide limited insight into the biological heterogeneity underlying disease onset and progression (Roach et al., 1993; Luks et al., 2017; Wang et al., 2024). Therefore, objective molecular markers and mechanistic frameworks for AMS remain insufficiently defined (Maggiorini et al., 2001; Wang et al., 2024; Li et al., 2025).

Beyond its relevance to high-altitude medicine, AMS provides a useful human model for investigating acute hypoxia-driven systemic remodeling. Hypoxia is also a central biological stressor in cardiovascular diseases and cancer, where it contributes to vascular dysfunction, endothelial activation, immune remodeling, coagulation disturbance, metabolic reprogramming, and altered tissue adaptation. These processes overlap with several mechanisms implicated in AMS, including oxidative stress, inflammatory activation, altered vascular permeability, fluid imbalance, and abnormal tissue responses to hypoxia (Pena et al., 2022; Gatterer et al., 2024; Zhang et al., 2025). Thus, studying AMS after rapid high-altitude exposure may provide broader insight into how acute hypoxic stress reshapes vascular, immune, and metabolic networks in humans.

High-throughput omics approaches offer an effective strategy for capturing the biochemical and regulatory disturbances associated with altitude maladaptation and acute hypoxic stress. Proteomics and metabolomics are particularly suited to this purpose because they reflect both upstream regulatory events and downstream functional consequences of hypoxia (Luo et al., 2014; Liao et al., 2016; He et al., 2020; Yang et al., 2022; Wu et al., 2025). Previous studies have reported plasma proteomic, transcriptomic, and clinical biomarkers associated with AMS susceptibility or prediction (Guo et al., 2023; Yan et al., 2024; Li et al., 2025; Yin et al., 2025). However, most available studies have focused on individual molecules or single-omics datasets, and a systematic framework integrating clinical phenotypes with coordinated proteomic and metabolomic alterations remains lacking. This limits both mechanistic interpretation and the development of precision biomarker models for AMS discrimination.

Here, we integrated plasma proteomic and metabolomic profiling with network analysis, tissue-specific protein mapping, machine learning, and druggability assessment to characterize the molecular architecture of AMS after rapid high-altitude exposure. This framework links AMS-associated molecular alterations with clinical phenotypes, tissue origin, upstream regulatory features, biomarker classification, and target prioritization, providing a basis for understanding hypoxia-driven immune–metabolic remodeling in humans.

## 2 Materials and methods

### 2.1 Study participants and cohort allocation

A total of 81 healthy Han Chinese male participants aged 18–45 years were enrolled in this study. The study protocol was approved by the Research Ethics Committee of Beijing Shijitan Hospital, Capital Medical University (approval No. ky2023-0092-001), and written informed consent was obtained from all participants.

Baseline demographic characteristics were recorded at low altitude before high-altitude exposure, and post-altitude physiological parameters were assessed after arrival at the high-altitude field site. All participants had no history of long-term residence at high altitude, defined as no stay above 2,500 m within the previous year, and had no chronic cardiovascular, respiratory, hepatic, renal, or neurological diseases. Following baseline assessment at low altitude (<500 m), all participants were transported by vehicle to the high-altitude field site (3,700 m) within 24 h. AMS was evaluated 6–48 h after arrival using the LLS, as summarized in Supplementary Table 1. AMS was defined as a total LLS ≥3 in the presence of headache.

The overall cohort was randomly divided into a training cohort and a test cohort at a 7:3 ratio. The training cohort comprised 56 participants (23 AMS and 33 non-AMS), whereas the test cohort comprised 25 participants (10 AMS and 15 non-AMS). The training cohort was used for feature selection and model development, and the test cohort was used for performance evaluation. To minimize analytical bias, samples from both cohorts were processed using the same experimental platform and analytical pipeline.

### 2.2 Clinical assessment and sample collection

At the high-altitude study site, trained research personnel recorded demographic and clinical information and performed LLS assessment for each participant. At the same time, peripheral venous blood samples were collected. Blood samples were centrifuged promptly after collection, and the separated plasma was aliquoted and stored at −80 °C until further analysis.

### 2.3 Multi-omics data acquisition and preprocessing

For proteomic analysis, plasma proteins were enriched using magnetic nanomaterials (PTM-Max; PTM Bio, Hangzhou, China), followed by reduction, alkylation, tryptic digestion, and C18 desalting. Peptides were separated on a Vanquish Neo ultra-high-performance liquid chromatography system and analyzed on an Orbitrap Astral mass spectrometer in Data Independent Acquisition (DIA). Raw data were processed in Spectronaut v18 with the built-in Pulsar search engine against the *Homo sapiens* database (updated 2 December 2024; 20,422 entries). Carbamidomethylation of cysteine was set as a fixed modification, whereas methionine oxidation and protein N-terminal acetylation were set as variable modifications. Up to two missed cleavages were allowed. Protein quantification was performed using the label-free quantification algorithm in Spectronaut, and the identification-level false discovery rate (FDR) was controlled at <1% at the peptide-spectrum match, peptide, and protein levels.

For metabolomic analysis, plasma samples were extracted with methanol:acetonitrile (1:1, v/v) containing isotopically labeled internal standards, and pooled quality control samples were prepared from equal aliquots of each extract. Metabolomic profiling was performed on a Vanquish ultra-high-performance liquid chromatography system coupled to an Orbitrap Exploris 120 mass spectrometer (Thermo Fisher Scientific, United States of America). Polar metabolites were separated on a Waters ACQUITY UPLC BEH Amide column, and non-polar metabolites were separated on a Phenomenex Kinetex C18 column. Raw data were subjected to peak extraction and alignment to generate a metabolite intensity matrix. Features with >50% missing values were excluded, and the remaining missing values were imputed using half of the minimum detected value. Data were normalized to the total ion current (TIC) of each sample. Multivariate analyses were performed in SIMCA (version 18.0.1; Sartorius Stedim Data Analytics AB, Umea, Sweden). After center scaling, principal component analysis (PCA) was used to assess overall sample distribution and potential outliers.

### 2.4 Statistical analysis

Statistical analyses were performed for both proteomic and metabolomic datasets. For proteomic analysis, differentially expressed proteins were defined as those with a fold change (FC) > 1.2 or <1/1.2 and P < 0.05. For metabolomic analysis, differential metabolites were identified using a combined univariate and multivariate strategy. Group differences were evaluated using Student's t-test, with P < 0.05 considered statistically significant. Orthogonal projections to latent structures-discriminant analysis (OPLS-DA) was then performed to evaluate intergroup separation, and metabolites with both P < 0.05 and variable importance in projection (VIP) > 1 were defined as significantly altered metabolites.

### 2.5 Bioinformatics and integrative multi-omics analysis

For the proteomic data, GO annotation was performed using eggNOG-mapper, and proteins were classified into biological process, molecular function, and cellular component categories. KEGG pathway annotation was conducted by BLASTP search (E-value ≤ 1e–4), and Reactome analysis was used for complementary pathway interpretation. Plasma proteins were further characterized according to tissue-specific protein features and cellular topology, including secreted and membrane-associated categories. Subcellular localization was predicted using WoLF PSORT. In addition, ssGSEA was performed to estimate pathway activity at the individual-sample level, and WGCNA was used to identify protein modules associated with clinical traits and disease phenotypes. Protein–protein interaction analysis was performed using the STRING database, and interactions with a confidence score >0.7 were visualized using the R package visNetwork.

For the metabolomic data, significantly altered metabolites were subjected to functional and pathway enrichment analyses, including GO and KEGG analyses, to characterize disturbed metabolic processes associated with AMS. WGCNA was further performed to identify metabolite modules associated with clinical traits and disease phenotypes, and metabolite modules of interest were subsequently selected for downstream functional interpretation.

For integrative multi-omics analysis, protein–metabolite co-expression analysis was performed to evaluate coordinated alterations between the proteome and metabolome. Correlation networks were constructed to identify functionally connected molecular signatures associated with AMS, and integrated functional interpretation was conducted to reveal biological pathways jointly dysregulated at the protein and metabolite levels.

### 2.6 Machine learning and model construction

A machine-learning workflow was applied for feature preprocessing, selection, model construction, and validation. Features with a missing rate of >50% were excluded, and the remaining missing values were imputed using the K-nearest neighbors (KNN) method. Features with zero variance were removed, and highly correlated features (Pearson’s r > 0.8) were filtered by retaining the variable with the strongest association with class labels. After preprocessing, the retained features were used for downstream analysis.

Multiple combinations of feature-selection strategies and supervised learning algorithms were evaluated in the training cohort. Among them, max-relevance and min-redundancy (mRMR) combined with logistic regression (LR) showed the best balance between discrimination and generalizability and was therefore selected as the final model. Model performance was subsequently assessed in the test cohort using sensitivity, specificity, accuracy, and the area under the receiver operating characteristic curve (AUC).

### 2.7 Drug-target association and druggability analysis

Core biomarkers with the greatest contribution to the final model were used as seed molecules for drug-target association and druggability analysis. These biomarkers were queried against publicly available databases, including DrugBank and other relevant drug-target resources, to identify known target–drug associations and to evaluate their druggability, such as whether they were targets of approved drugs or had reported bioactive compounds. This analysis was performed to prioritize candidate biomarkers with potential translational relevance.

## 3 Results

### 3.1 Physiological differences between AMS and non-AMS participants after rapid high-altitude exposure

Following rapid high-altitude exposure, AMS participants showed significantly lower SpO2 and higher rate-pressure product (RPP) than non-AMS participants, whereas heart rate, systolic blood pressure, diastolic blood pressure, and mean arterial pressure did not differ significantly between groups (Table 1).

**TABLE 1:** Baseline demographic characteristics and post-altitude physiological parameters in the non-AMS and AMS groups.

Parameter	non-AMS (n=48)	AMS (n=33)	*P* value
Demographic characteristics			
Age (years)	21.00 (20.00, 22.75)	21.00 (20.00, 22.00)	0.30
Body mass index (kg/m^2^)	22.15 (20.67, 23.85)	22.76 (22.23, 24.51)	0.44
Physiological parameters			
Peripheral oxygen saturation (%)	85.0 (84.0, 86.0)	84.0 (79.5, 86.0)	0.01
Heart rate (bpm)	91.85 ± 11.97	96.79 ± 13.17	0.07
Systolic blood pressure (mmHg)	129.38 ± 13.42	134.58 ± 15.00	0.11
Diastolic blood pressure (mmHg)	77.90 ± 9.48	80.70 ± 9.91	0.20
Mean arterial pressure (mmHg)	95.06 ± 9.83	98.66 ± 11.23	0.13
Rate-pressure product (bpm·mmHg)	119.37 ± 22.81	130.96 ± 25.58	0.04

### 3.2 Multi-omics profiling reveals extensive molecular dysregulation in AMS

Plasma multi-omics profiling revealed marked molecular differences between the AMS and non-AMS groups. OPLS-DA showed separation between groups in both the proteomic and metabolomic datasets (Supplementary Figure S1). Across all samples, 21,111 peptides mapping to 3,137 proteins and 4,104 metabolites were identified.

### 3.3 Proteomic alterations indicate extracellular remodeling and functional reprogramming in AMS

**FIGURE 1:**
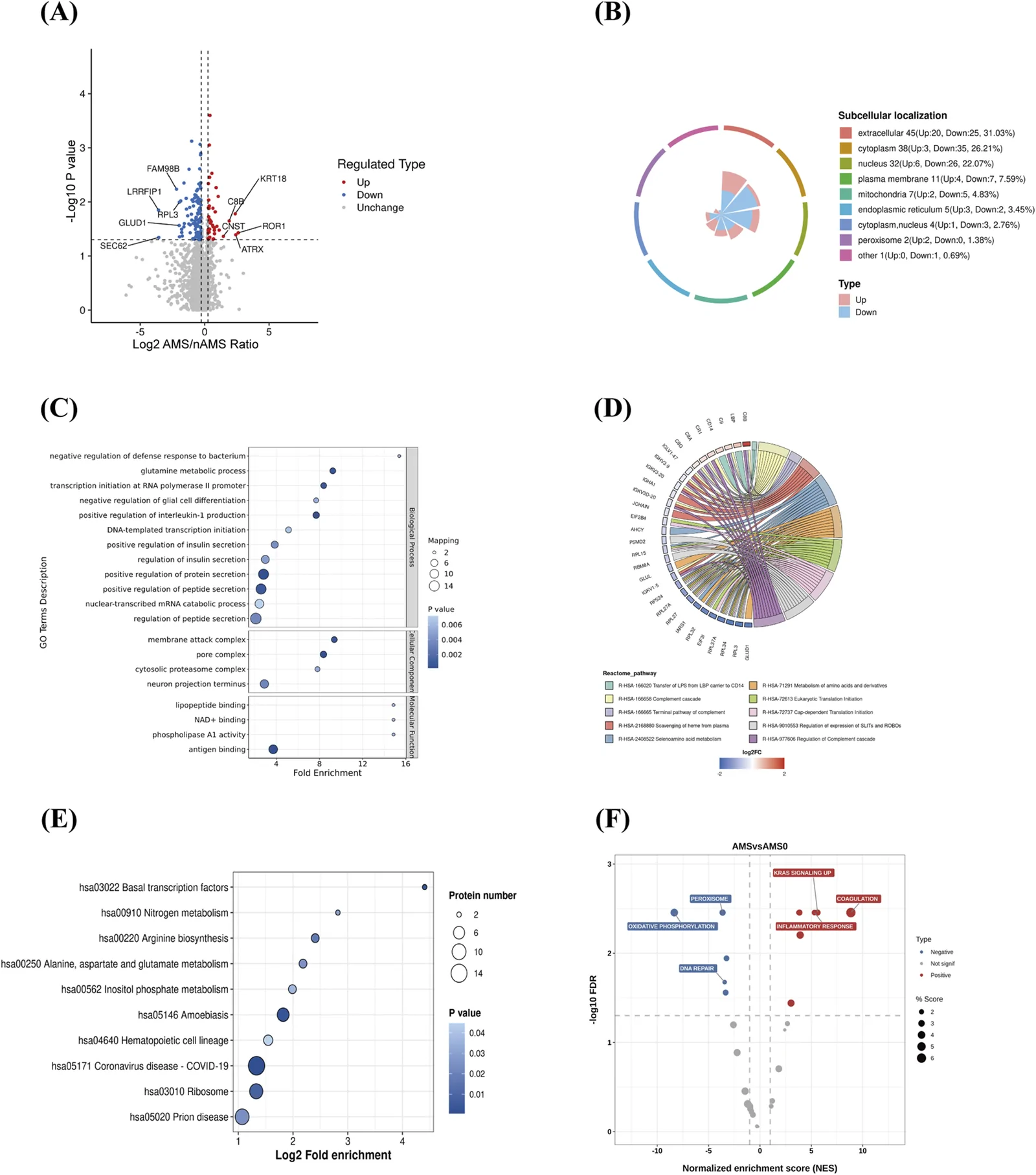
Global multi-omics profiling and proteomic functional characterization in AMS **(A)** Volcano plot of differentially expressed proteins (DEPs) between the AMS and non-AMS groups. Red dots indicate upregulated proteins, blue dots indicate downregulated proteins, and gray dots indicate proteins without significant change **(B)** Rose plot showing the subcellular localization distribution of DEPs. Upregulated and downregulated proteins are shown separately within each subcellular compartment **(C)** Gene Ontology (GO) enrichment analysis of DEPs, including biological process, cellular component, and molecular function categories. Bubble size represents the number of enriched proteins, and color indicates the enrichment P value **(D)** Reactome pathway analysis of DEPs. The plot shows the relationships between DEPs and significantly enriched Reactome pathways **(E)** KEGG pathway enrichment analysis of DEPs. Bubble size represents the number of enriched proteins, and color indicates the enrichment P value **(F)** Hallmark-based single-sample gene set enrichment analysis (ssGSEA) showing altered functional signatures in the AMS group relative to the non-AMS group. Red and blue dots indicate positively and negatively enriched pathways, respectively.

Using the predefined criteria, we identified 145 differentially expressed proteins (DEPs) between the AMS and non-AMS groups, including 41 upregulated and 104 downregulated proteins in AMS (Figure 1A).

Subcellular localization analysis showed that the largest proportion of DEPs originated from the extracellular space (45 proteins, 31.0%), followed by the cytoplasm (38, 26.2%) and nucleus (32, 22.1%) (Figure 1B). Mitochondrial proteins accounted for 4.8% of the differential proteome.

Functional enrichment analyses further characterized the biological distribution of these DEPs. GO analysis showed enrichment in cellular metabolism, developmental regulation, and responses to stress and stimuli, whereas molecular function terms were enriched for binding and catalytic activities (Figure 1C). KEGG analysis highlighted basal transcription factors, nitrogen metabolism, and arginine biosynthesis (Figure 1E), while Reactome analysis linked the DEPs to the complement cascade, transfer of LPS from LBP carrier to CD14 (Figure 1D).

To further determine whether these pathway-level changes converged into broader biological states, we performed ssGSEA at the individual-sample level (Figure 1F). Compared with the non-AMS group, AMS samples showed significant upregulation of KRAS signaling, coagulation, and inflammatory response signatures, together with marked downregulation of peroxisome, oxidative phosphorylation, and DNA repair signatures.

### 3.4 Metabolomic perturbations and multi-omics integration

**FIGURE 2:**
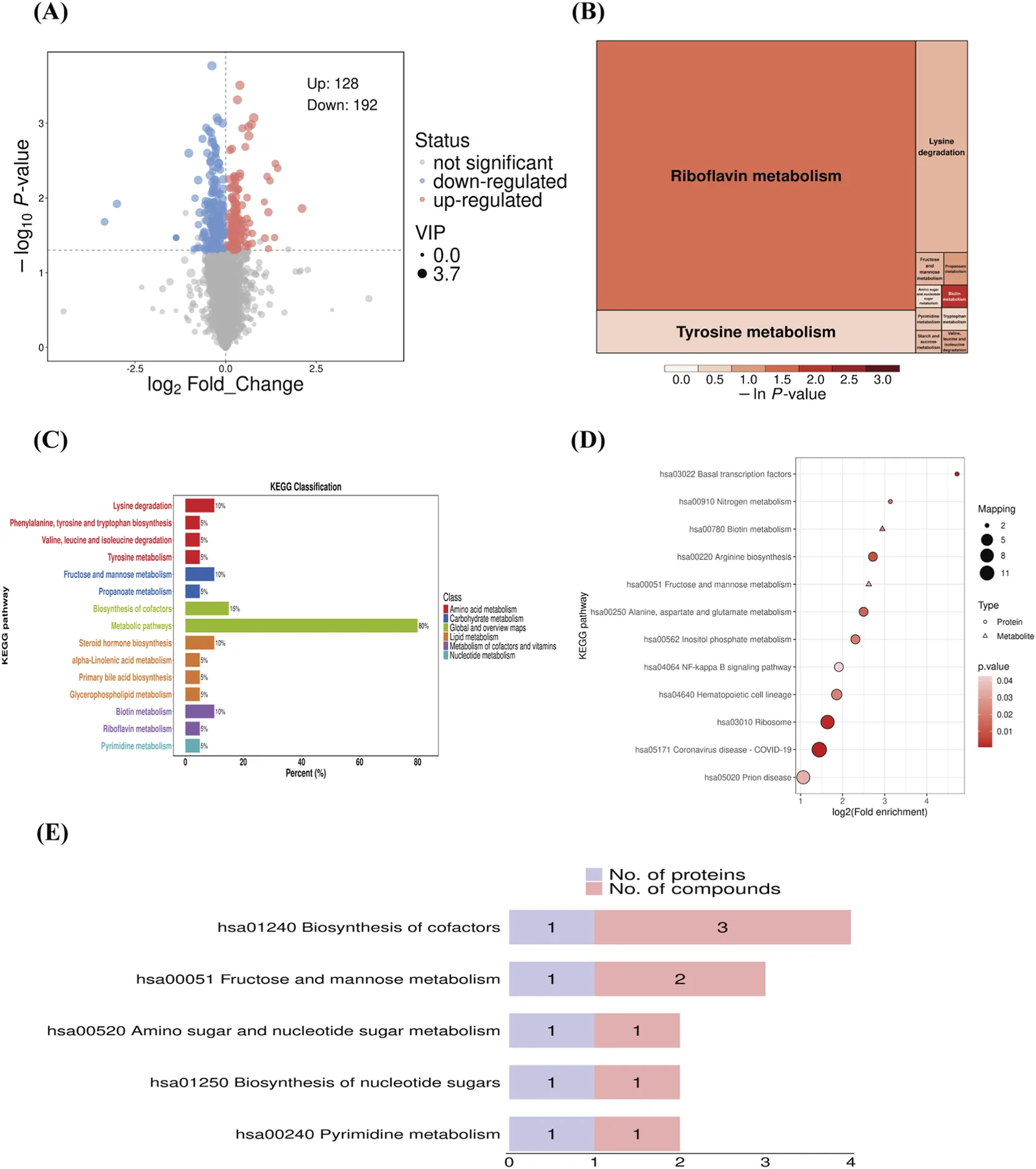
Metabolomic alterations and metabolite–protein association analysis in AMS **(A–C)** Metabolomic analysis of the AMS and non-AMS groups **(A)** Volcano plot of differential metabolites. Red dots indicate upregulated metabolites, blue dots indicate downregulated metabolites. Dot size represents VIP value **(B)** Treemap showing significantly enriched metabolic pathways associated with differential metabolites. Color intensity reflects the enrichment significance **(C)** KEGG classification of differential metabolites. Bars represent the proportion of metabolites assigned to each KEGG pathway category **(D,E)** Association analysis between differential metabolites and differential proteins **(D)** KEGG pathway mapping analysis showing pathways jointly involved in the proteomic and metabolomic alterations. Purple bars indicate the numbers of differential proteins mapped to each pathway, and red bars indicate the numbers of differential metabolites mapped to each pathway **(E)** KEGG enrichment analysis based on the integrated differential proteins and metabolites. Triangles represent metabolites, circles represent proteins, bubble size indicates the number of mapped molecules, and color indicates the enrichment P value.

Differential metabolite analysis identified 320 altered metabolites, including 128 upregulated and 192 downregulated metabolites in the AMS group (Figure 2A). These metabolites were mainly involved in amino acid metabolism, carbohydrate metabolism, lipid metabolism, nucleotide metabolism, and the metabolism of cofactors and vitamins (Figures 2B,C). Biosynthesis of cofactors, fructose and mannose metabolism, lysine degradation, and biotin metabolism showed the strongest enrichment signals.

To further investigate cross-omics convergence, we integrated the proteomic and metabolomic datasets and identified pathways jointly represented by differential proteins and metabolites. Five shared pathways were detected, including biosynthesis of cofactors, fructose and mannose metabolism, amino sugar and nucleotide sugar metabolism, biosynthesis of nucleotide sugars, and pyrimidine metabolism (Figures 2D,E). Enrichment analysis of the integrated differential molecules further highlighted NF-κB signaling, basal transcription factors, nitrogen metabolism, and ribosome-related processes.

### 3.5 WGCNA identifies symptom-associated proteomic and metabolomic modules in AMS

**FIGURE 3:**
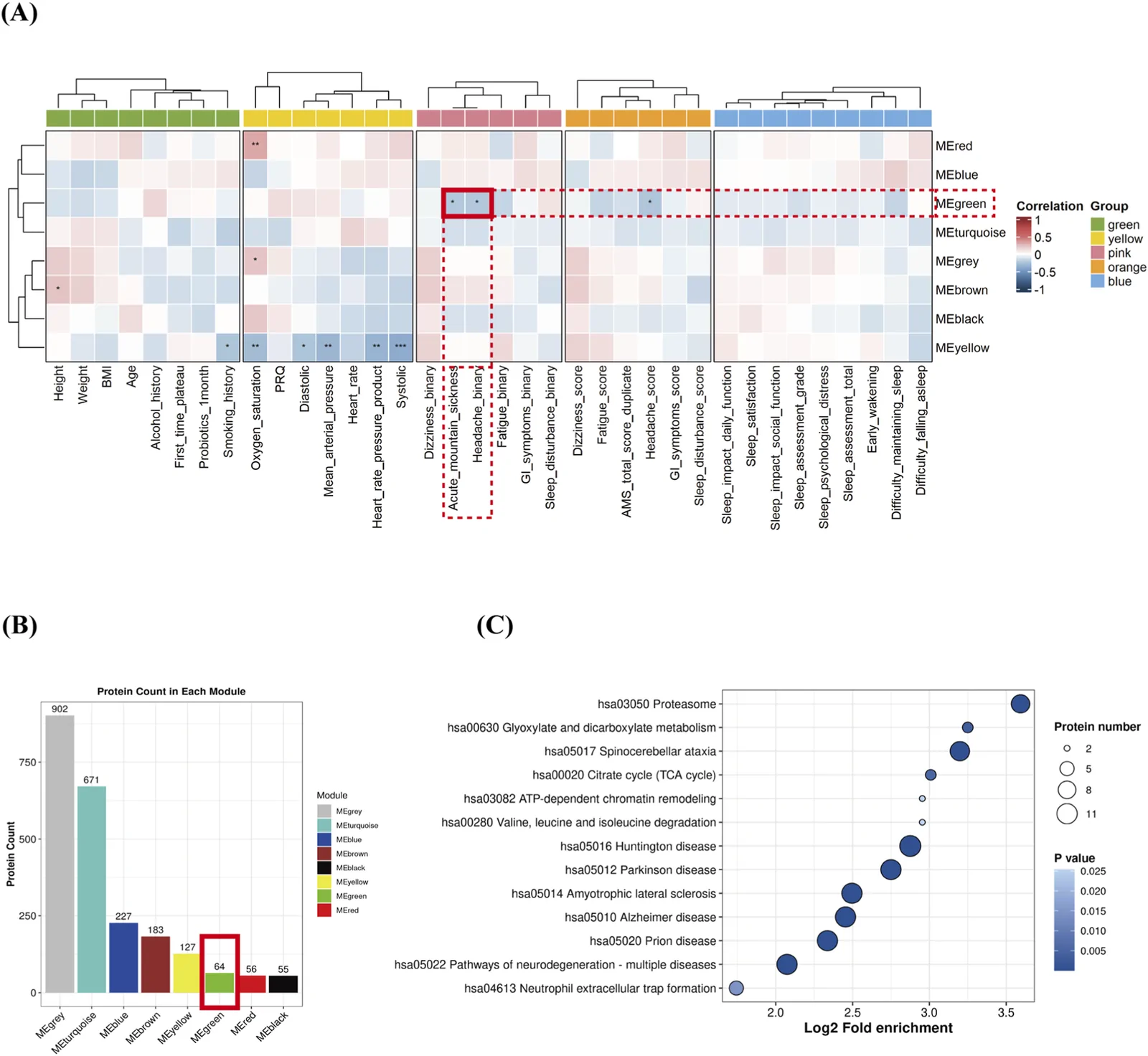
WGCNA identifies clinically relevant proteomic modules associated with AMS **(A)** Heatmap of correlations between protein modules and clinical phenotypes. Color indicates the correlation coefficient, and asterisks indicate statistically significant associations **(B)** Bar plot showing the numbers of proteins in each co-expression module **(C)** KEGG enrichment analysis of proteins in the clinically relevant module. Bubble size represents the number of enriched proteins, and color indicates the enrichment P value.

**FIGURE 4:**
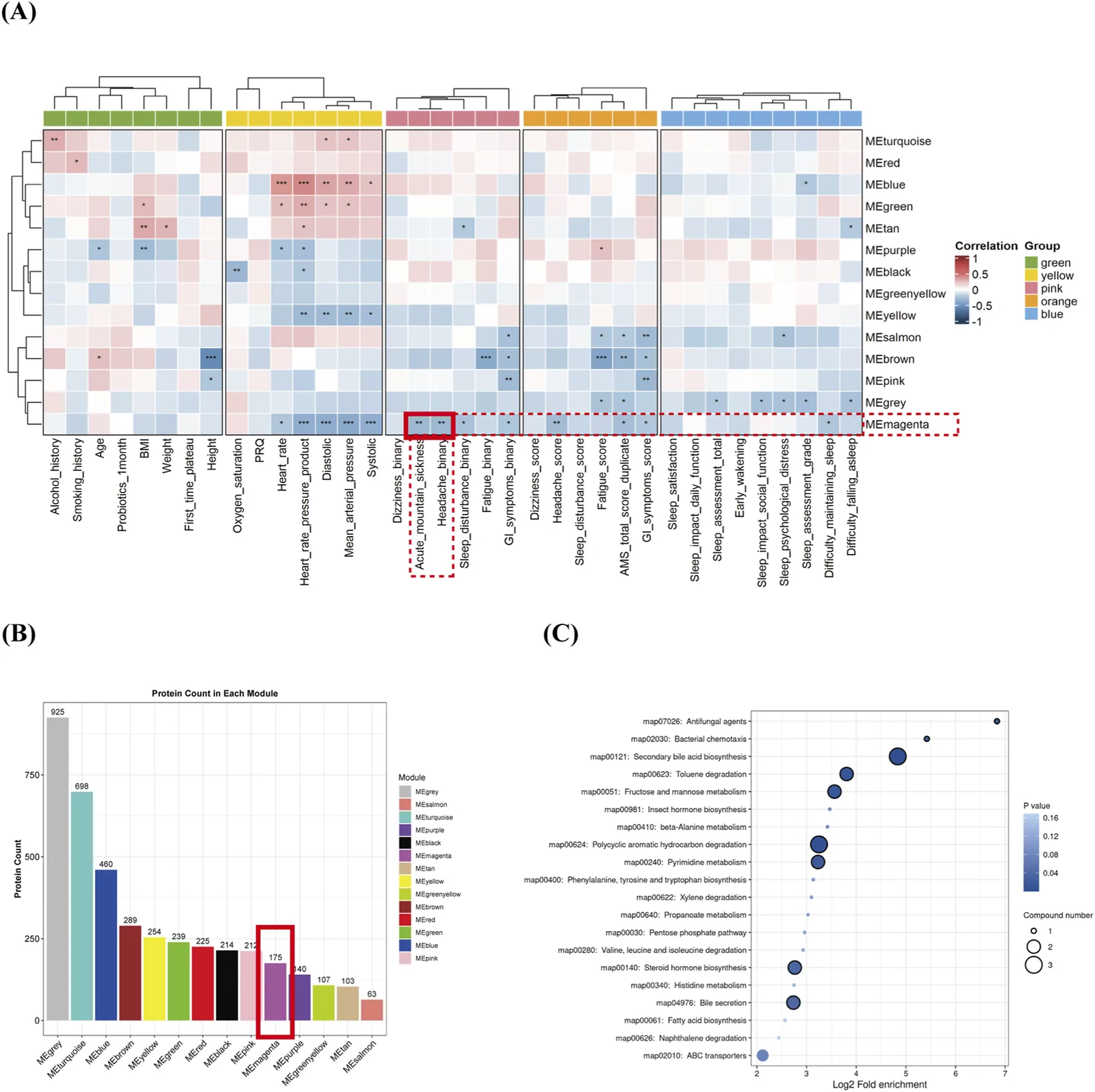
WGCNA identifies clinically relevant metabolomic modules associated with AMS **(A)** Heatmap of correlations between metabolomic modules and clinical phenotypes. Color indicates the correlation coefficient, and asterisks indicate statistically significant associations **(B)** Bar plot showing the numbers of metabolites in each co-abundance module **(C)** KEGG enrichment analysis of metabolites in the magenta module, which showed the strongest association with AMS-related phenotypes.

WGCNA was performed on the proteomic and metabolomic datasets to identify phenotype-associated molecular modules. Sample hierarchical clustering revealed no obvious outliers, indicating good data quality for network construction. A soft-thresholding power of 20 was selected to achieve approximate scale-free topology, with a corresponding R^2^ = 0.9 and high mean connectivity. Using hierarchical clustering and dynamic tree cutting, we identified 8 proteomic modules and 14 metabolomic modules (Figures 3, 4; Supplementary Figures S2, S3).

In the proteomic dataset, several co-expression modules showed distinct associations with clinical traits (Figure 3A). The yellow module (127 proteins) displayed the broadest correlations with key physiological parameters, including systolic blood pressure, RPP, mean arterial pressure, and peripheral oxygen saturation (r = −0.28 to −0.38, P < 0.05). The red module showed the strongest positive association with oxygen saturation (r = 0.32, P = 0.004). Notably, the green module (64 proteins) exhibited the strongest negative correlation with AMS status and headache severity (r ≈ −0.24, P < 0.05), indicating particular clinical relevance in this cohort (Figure 3B). KEGG enrichment analysis (Figure 3C) of the green-module proteins revealed significant enrichment in the citrate cycle, proteasome, neutrophil extracellular trap formation, and valine, leucine and isoleucine degradation pathways.

In the metabolomic dataset, module–trait analysis identified the magenta module (175 metabolites) as the metabolite module most strongly associated with AMS status (r = −0.31, P = 0.004) (Figure 4A,B). This module was also significantly correlated with blood pressure and rate-pressure product (r = −0.36 to −0.40, P = 0.004 to 0.001), suggesting close links to cardiovascular and hemodynamic responses under hypoxic stress. KEGG enrichment analysis (Figure 4C) further showed that the magenta-module metabolites were mainly enriched in steroid hormone biosynthesis, amino acid metabolism, fructose and mannose metabolism, nucleotide metabolism, and the pentose phosphate pathway.

### 3.6 Tissue-specific protein mapping reveals a liver-centered but multi-organ molecular architecture in AMS

**FIGURE 5:**
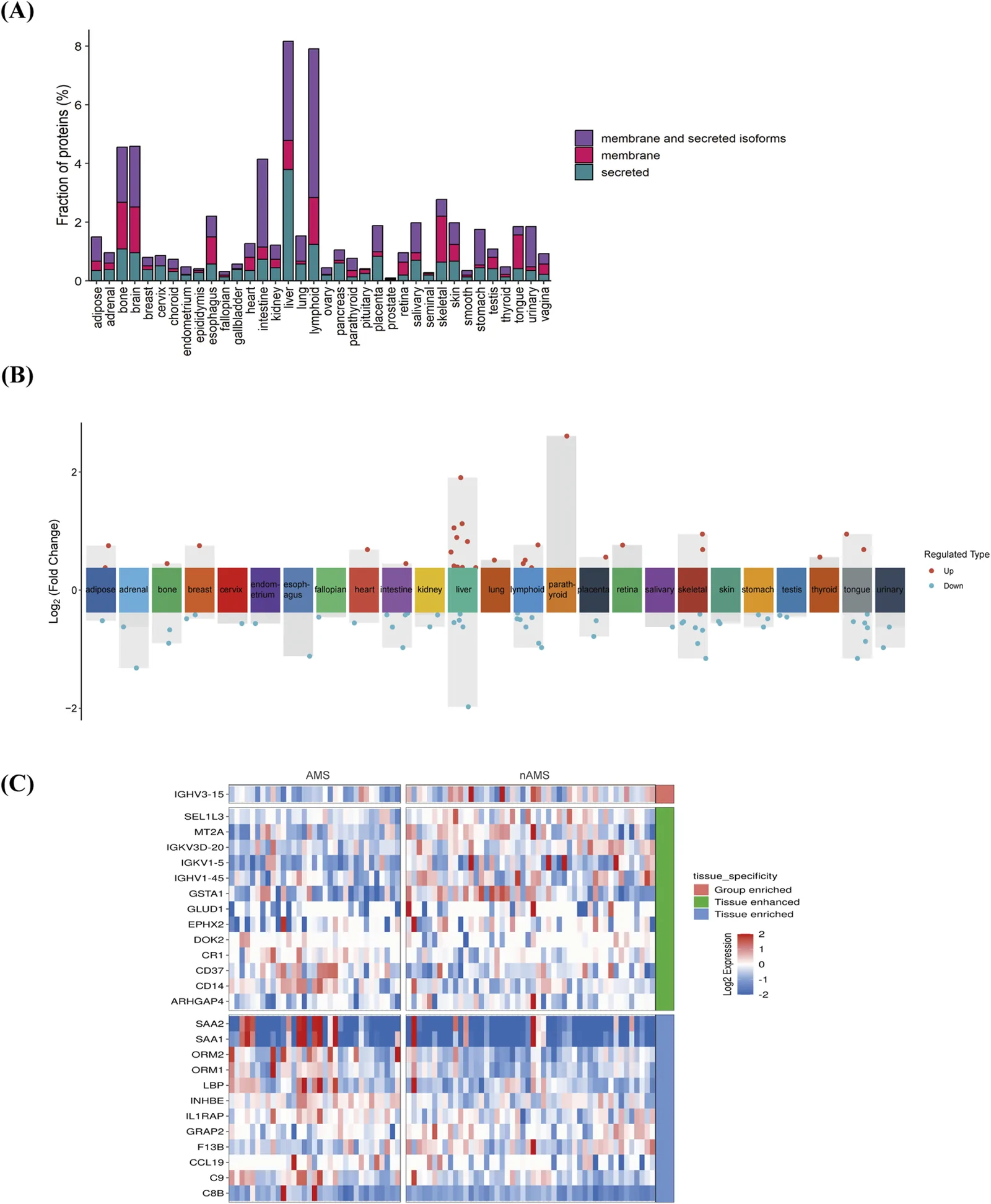
Tissue-specific protein mapping reveals liver-centered circulating proteomic remodeling in AMS **(A)** Global tissue-specific mapping of the overall identified plasma proteins. Proteins were classified according to cellular topology as secreted, membrane-associated, or membrane/secreted dual isoforms, and their tissue distribution is shown as the fraction of proteins assigned to each tissue category **(B)** Organ localization of differentially expressed proteins (DEPs) between the AMS and non-AMS groups. Each dot represents a DEP mapped to a tissue category, and the y-axis indicates the log2 (fold change). Red dots indicate upregulated proteins and blue dots indicate downregulated proteins **(C)** Heatmap of representative tissue-specific DEPs in the AMS and non-AMS groups. Colors represent relative protein abundance, and side annotations indicate tissue-specific categories.

To further investigate the anatomical origin of the circulating proteome in AMS, we first mapped the overall identified plasma proteins to tissue-specific protein profiles and further classified them according to cellular topology as secreted, membrane-associated, or dual-localized proteins (Figure 5). The circulating proteome was predominantly enriched for liver-derived proteins, with additional contributions from lymphoid tissues, the brain, bone, and skeletal muscle (Figure 5A). Liver- and intestine-associated proteins were mainly classified as secreted proteins, whereas brain- and lymphoid-associated proteins were more frequently membrane-associated.

Tissue-specific mapping of the clinically relevant WGCNA module also showed predominant enrichment of liver-derived secreted proteins (Supplementary Figure S4). At the DEPs level, tissue mapping showed a predominant hepatic origin (Figure 5B). Representative liver-associated DEPs included upregulated acute-phase and host-defense proteins, such as SAA1/2, ORM1/2, and LBP, together with downregulated GSTA1 (Figure 5C).

### 3.7 Regulatory network analysis suggests upstream coordination of the systemic proteomic response in AMS

**FIGURE 6:**
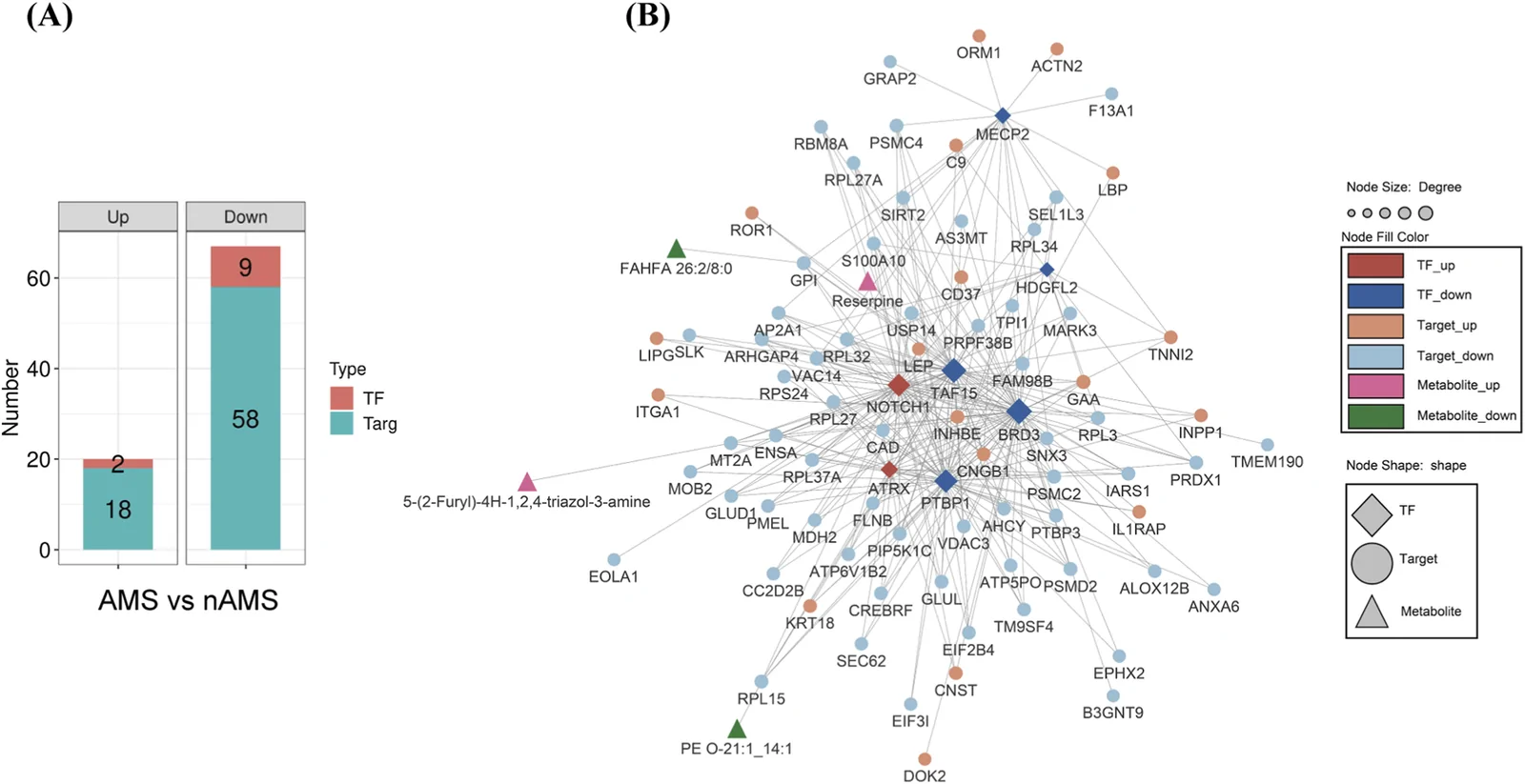
Upstream regulatory network analysis of AMS-associated molecular alterations **(A)** Bar plot showing the numbers of differential regulatory factors and their downstream target proteins in the AMS versus non-AMS comparison **(B)** Transcription factor–target protein–metabolite regulatory network. Diamonds represent transcription factors, circles represent target proteins, and triangles represent metabolites. Node colors indicate the direction of expression change, Node size indicates the degree of connectivity.

To identify upstream regulatory hubs potentially coordinating the systemic molecular response in AMS, differentially expressed proteins were mapped to the TRRUST and GTRD databases to reconstruct a regulatory factor–target protein network (Figure 6; Table 2). The resulting network showed a predominantly downregulated pattern, with 9 downregulated regulatory factors and 58 downregulated target proteins, compared with 2 upregulated regulatory factors and 18 upregulated target proteins. Representative differential regulatory factors and their putative functions are summarized in Table 2. The differential regulatory factors were mainly related to chromatin remodeling, transcriptional control, RNA processing, and inflammatory signaling.

**TABLE 2:** Differential regulatory factors identified in the AMS-associated regulatory network.

Regulatory factor	FC	*P* value	Putative function
YBX3	0.754	0.043	Member of the cold-shock protein family; binds and stabilizes mRNA to regulate translation
PTBP1	0.569	0.025	RNA-binding protein involved in mRNA splicing, stability, and IRES-mediated translation
ATRX	5.336	0.041	Core component of the chromatin-remodeling complex; maintains telomere and heterochromatin stability
NOTCH1	1.569	0.029	Key transmembrane receptor controlling cell fate; regulates inflammation, proliferation, and vascular development
MECP2	0.610	0.028	Methyl-CpG-binding protein acting as a transcriptional repressor to maintain neuronal homeostasis
GTF2I	0.460	0.037	General transcription factor involved in immune responses and growth factor signaling
BRD3	0.695	0.039	Acetylated histone reader that promotes RNA polymerase II transcriptional elongation
LRRFIP1	0.084	0.014	Transcriptional regulator involved in innate immune and inflammatory signaling, including TNF-α-related pathways
HDGFL2	0.605	0.032	Chromatin-associated protein involved in transcriptional regulation and cell survival-related signaling
TAF15	0.637	0.037	FET family RNA-binding protein involved in transcription initiation and stress granule formation
THRAP3	0.577	0.049	Nuclear protein involved in pre-mRNA splicing and nuclear export

### 3.8 Machine learning identifies a protein–physiological classifier for AMS

**FIGURE 7:**
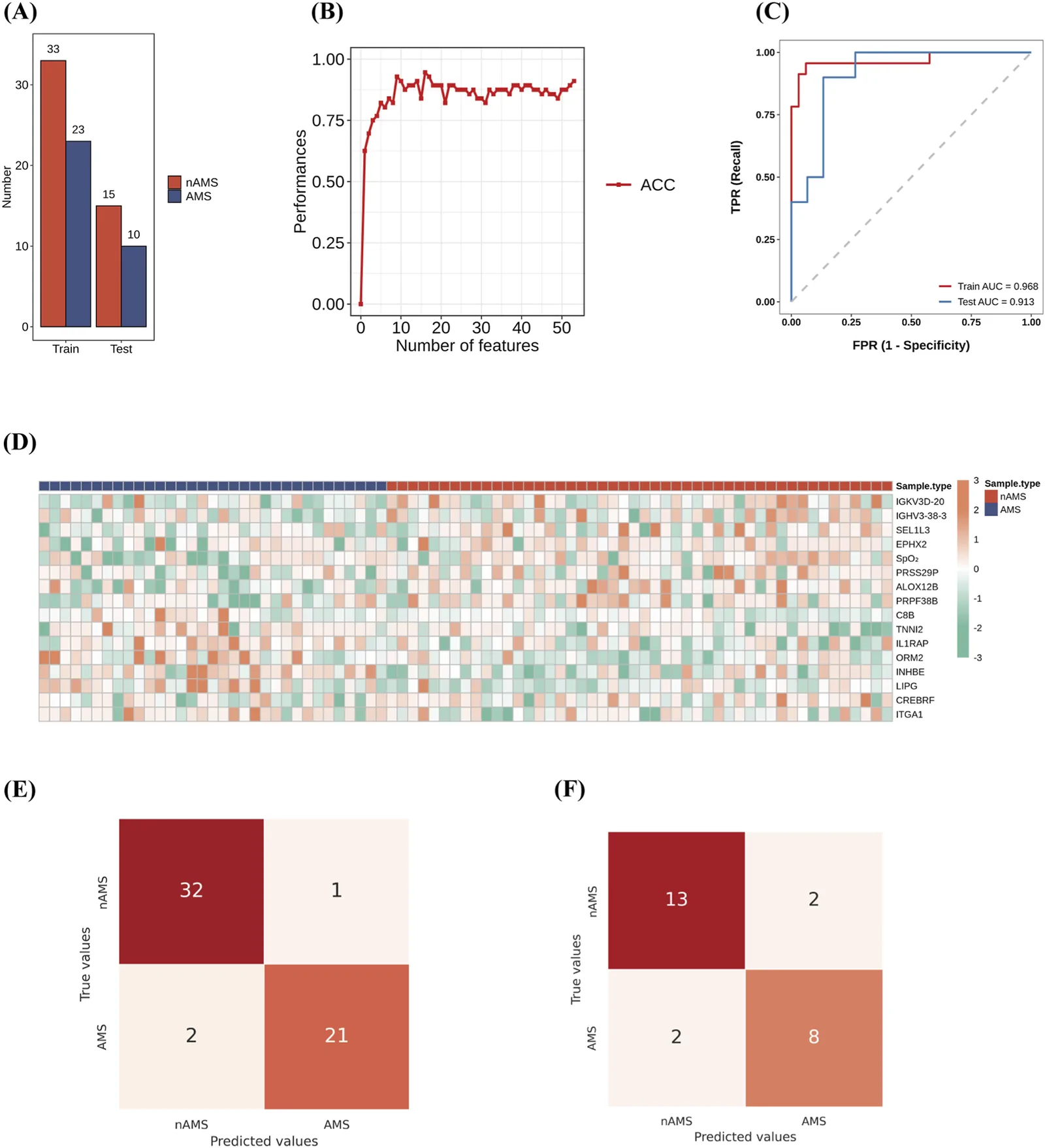
Construction and performance of the final mRMR + LR classifier for AMS **(A)** Distribution of AMS and non-AMS samples in the discovery/training and test cohorts **(B)** Classification performance across different numbers of selected features during model optimization **(C)** Receiver operating characteristic (ROC) curves of the final mRMR + LR classifier in the training and test cohorts **(D)** Heatmap of the 16 selected features included in the final model. Colors represent relative feature abundance, and the top annotation indicates sample type **(E)** Confusion matrix of the classifier in the training cohort **(F)** Confusion matrix of the classifier in the test cohort.

To identify the optimal classifier for AMS, we evaluated multiple combinations of feature-selection strategies and supervised learning algorithms. Among the tested models, mRMR + LR showed the best balance between discrimination and generalizability and was therefore selected as the final classifier (Figure 7).

Based on the feature set selected by mRMR, we constructed a logistic regression model using proteomic and physiological variables (Figures 7A,B). The final model included 16 features, comprising 1 physiological parameter (SpO_2_) and 15 proteomic variables (Table 3).

**TABLE 3:** Physiological and proteomic features included in the final mRMR + LR model for AMS classification.

Variable	Full name/description
Physiological parameter	
SpO_2_	Peripheral oxygen saturation
Proteomic feature	
ALOX12B	Arachidonate 12-lipoxygenase, 12R-type
C8B	Complement C8 beta chain
ORM2	Orosomucoid 2
EPHX2	Epoxide hydrolase 2
TNNI2	Troponin I2, fast skeletal type
ITGA1	Integrin subunit alpha 1
CREBRF	CREB3 regulatory factor
IL1RAP	Interleukin 1 receptor accessory protein
LIPG	Lipase G, endothelial type
INHBE	Inhibin subunit beta E
IGKV3D-20	Immunoglobulin kappa variable 3D-20
PRSS29P	Putative serine protease 29
IGHV3-38–3	Immunoglobulin heavy variable 3–38-3
PRPF38B	Pre-mRNA processing factor 38B
SEL1L3	SEL1L family member 3

In the training cohort (23 AMS and 33 non-AMS participants), the model achieved an AUC of 0.968, with a specificity of 0.970 and a sensitivity of 0.950. In the test cohort (10 AMS and 15 non-AMS participants), the model maintained good predictive performance, with an AUC of 0.913, a specificity of 0.867, and a sensitivity of 0.840 (Figures 7C,D,E,F).

### 3.9 Drug-target association and druggability analysis prioritizes candidate therapeutic targets in AMS

**FIGURE 8:**
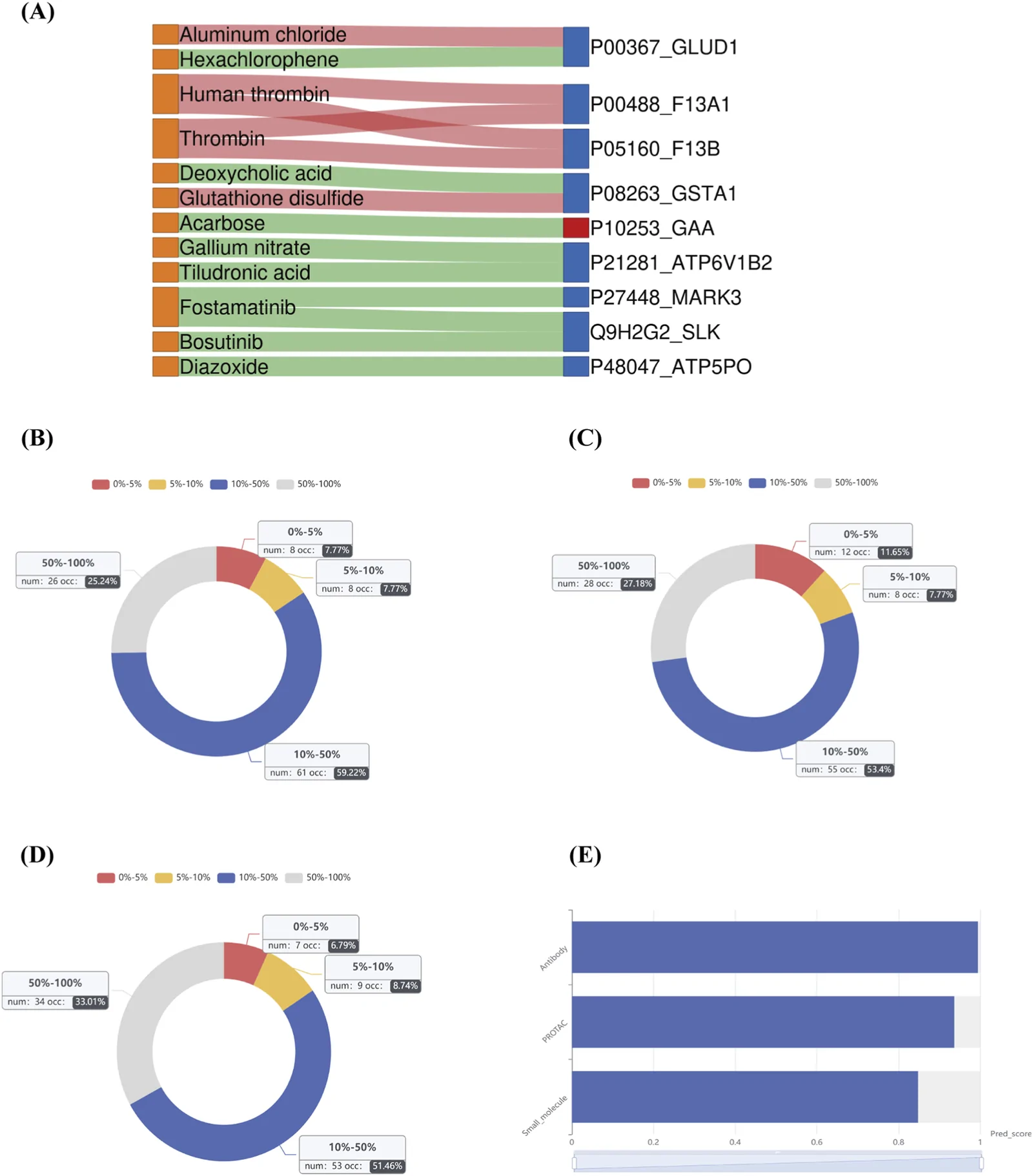
Drug–target associations and druggability prioritization of candidate therapeutic targets in AMS **(A)** Drug–target Sankey diagram showing known associations between FDA-approved agents and dysregulated proteins identified in AMS **(B–D)** Doughnut plots showing the distributions of predicted druggability probability scores for antibody-based, PROTAC-based, and small-molecule modalities, respectively **(E)** Predicted druggability scores of NOTCH1 for antibody-based, PROTAC-based, and small-molecule modalities.

To assess the translational relevance of AMS-associated proteins, key biomarkers were mapped to the DrugBank database to identify known drug–target associations. The resulting Sankey diagram (Figure 8A) revealed multiple links between FDA-approved agents and dysregulated proteins. Directionally consistent associations were observed for glutathione disulfide–GSTA1 and acarbose–GAA, suggesting potential relevance for drug repurposing. Notably, GSTA1 is of particular interest because it represents a convergence point between model selection, antioxidant biology, and pharmacological tractability.

We next prioritized NOTCH1, identified as a differential upstream regulatory factor, for druggability evaluation. Multi-modal prediction (Figure 8E) showed high predicted druggability of NOTCH1 across several therapeutic modalities, with the highest score for antibody-based targeting (>0.99), followed by PROTACs (0.94) and small molecules (0.85). These findings support the prioritization of NOTCH1 and GSTA1 for future functional validation.

## 4 Discussion

### 4.1 Principal findings

AMS represents an acute clinical manifestation of hypobaric hypoxia and provides a window into systemic hypoxia-related remodeling (Murdoch, 1999; Moore, 2001; Gatterer et al., 2024). In this study, integrated proteomic and metabolomic analyses showed that AMS was associated with inflammatory activation, coagulation-related disturbance, metabolic disorder, and liver-centered proteomic remodeling. Network-based analyses further linked these molecular alterations to AMS status, headache severity, oxygen saturation, and hemodynamic traits. These findings extend AMS research beyond symptom scoring and single-layer biomarker discovery, providing a systems-level view of, immune, metabolic, and tissue-origin features of acute hypobaric hypoxia.

### 4.2 Thrombo-inflammatory activation and metabolic disorder in AMS

One of the central findings of this study is the coexistence of thrombo-inflammatory activation and metabolic disorder in AMS. Complement-related signaling, coagulation-associated pathways, and altered central carbon metabolism were accompanied by broad metabolomic disturbances involving amino acid, carbohydrate, nucleotide, lipid, and steroid-related pathways. At the pathway-activity level, ssGSEA further showed enrichment of inflammatory and coagulation signatures, together with suppression of oxidative phosphorylation- and peroxisome-related functions. This pattern is consistent with previous reports showing that acute high-altitude exposure enhances inflammatory signaling and that oxidative stress is an important component of altitude-related illness (Pena et al., 2022; Pham et al., 2022; Yang et al., 2022). Notably, this pattern shares several features with hypoxia-related remodeling in cardiovascular disease and tumor microenvironments, including vascular stress, immune activation, coagulation disturbance, and metabolic reprogramming (Wu et al., 2022; Semenza, 2023; Zhao et al., 2023). The present study extends these observations by demonstrating that these processes are coordinated across proteomic and metabolomic layers and are linked to clinical phenotypes within the same analytical framework.

The absence of directly detectable HIF proteins in plasma does not contradict the classical hypoxia-response paradigm. HIF proteins are intracellular, dynamically regulated, and typically present at low circulating abundance; therefore, their absence from plasma proteomic profiles is not unexpected (Berra et al., 2001; Semenza, 2012; Zhang et al., 2025). In this context, downstream inflammatory, coagulation-related, and metabolic signatures may serve as more accessible peripheral readouts of systemic hypoxic stress (Luo et al., 2022). The identification of NOTCH1 as an altered upstream regulatory factor is also noteworthy. Notch signaling is known to interact with HIF-dependent pathways and to participate in angiogenesis, endothelial regulation, inflammation, and metabolic adaptation (Gustafsson et al., 2005; Fazio and Ricciardiello, 2016; O'Brien et al., 2022; Li et al., 2024; Oliveira et al., 2024). Thus, NOTCH1 may represent a candidate regulatory node in the systemic response to acute hypoxia, although its causal role in AMS requires further experimental validation.

### 4.3 Tissue-origin features of the circulating proteome

A distinctive aspect of this study is the tissue-origin analysis of the circulating proteome. The overall plasma proteome was predominantly enriched for liver-derived proteins, with additional contributions from lymphoid tissues, the brain, bone, and skeletal muscle. This tissue-origin pattern was accompanied by distinct protein topology. Liver- and intestine-associated proteins were mainly secreted, whereas brain- and lymphoid-associated proteins were more often membrane-associated. Given the sensitivity of the nervous and immune systems to hypoxia, these membrane-associated signals may reflect their involvement in the circulating molecular response to acute hypobaric hypoxia (Julian et al., 2011).

The clinically relevant WGCNA protein module further emphasized this liver-centered pattern, as it was predominantly composed of liver-derived secreted proteins. At the level of differentially expressed proteins, several liver-associated acute-phase and host-defense proteins, including SAA1/2, ORM1/2, and LBP, were upregulated, consistent with activation of hepatic acute-phase and innate immune responses (Gabay and Kushner, 1999), whereas the antioxidant enzyme GSTA1 was downregulated. Previous proteomic studies have implicated inflammatory and immune-related biomarkers in AMS (Yang et al., 2022; Guo et al., 2023; Li et al., 2025). Our findings are consistent with these reports but further suggest that the circulating response in AMS is structurally organized around hepatic secretory remodeling, with concurrent neural and immune-related contributions. This inter-organ perspective may help explain why AMS manifests as a systemic syndrome rather than a purely neurological or respiratory response to hypoxia.

### 4.4 Biomarker modeling and translational prioritization

The final mRMR + LR classifier, constructed from 15 proteomic features and SpO_2_, showed good discriminatory performance in both the training and test cohorts. This finding supports the view that AMS-related information is distributed across multiple biological layers rather than concentrated in a single marker class. Previous studies have shown that plasma proteomics, transcriptomics, and multidimensional phenotype-based modeling can yield useful AMS biomarkers and predictive models (Yan et al., 2024; Li et al., 2025; Yang et al., 2025). The present study differs by embedding molecular classification within a broader biological framework that also incorporates tissue origin, upstream regulation, and downstream target prioritization.

The translational implications of these findings should be interpreted in the context of target prioritization rather than immediate therapeutic application. DrugBank mapping revealed known associations between several dysregulated proteins and approved agents, and some of these associations were directionally consistent with the observed expression changes. Among these candidates, GSTA1 is of particular interest because it was downregulated in AMS and is closely related to antioxidant defense (Liang et al., 2005). At a broader regulatory level, NOTCH1 combined upstream relevance with high predicted druggability across antibody-, PROTAC-, and small-molecule-based modalities. Although these observations do not establish therapeutic efficacy, they support the prioritization of GSTA1 and NOTCH1 as candidate molecules for future functional validation in hypoxia-associated systemic remodeling.

### 4.5 Limitations

Several considerations should be noted. First, although the cohort was sufficient for an exploratory multi-omics analysis of AMS, the sample size was modest, and the classifier was evaluated using an internal test cohort rather than an external validation cohort. Second, the study population was relatively homogeneous, and validation in more diverse populations will be needed to assess generalizability. Third, the differential omics findings remain discovery-stage results and require targeted validation. Finally, tissue-origin mapping, regulatory network analysis, and druggability assessment were mainly hypothesis-generating and should be confirmed by functional studies.

### 4.6 Conclusion

In conclusion, this study defines AMS as a systemic molecular state characterized by thrombo-inflammatory activation, metabolic disorder, liver-centered secretory remodeling, and candidate upstream regulatory disturbance. By integrating proteomics, metabolomics, WGCNA, machine learning, and druggability assessment, we identified molecular signatures and candidate targets that may support future biomarker validation and mechanistic studies of acute hypobaric hypoxia. These findings provide a systems-level framework for understanding AMS and highlight the potential value of multi-omics strategies for precision risk assessment.

## Data Availability

The datasets presented in this study can be found in online repositories. The names of the repository/repositories and accession number(s) can be found below: http://www.proteomexchange.org/,PXD078227.

## Supplementary material

SUPPLEMENTARY FIGURE 1 Multi-omics OPLS-DA score plots of AMS and non-AMS participants. **(A)** OPLS-DA score plot based on metabolomic data. **(B)** OPLS-DA score plot based on proteomic data.

SUPPLEMENTARY FIGURE 2 Network construction and module detection in proteomic WGCNA. **(A)** Hierarchical clustering dendrogram of all samples used for WGCNA. **(B)** Analysis of the soft-thresholding power for network construction. The left panel shows the scale-free topology fit index, and the right panel shows the mean connectivity under different soft-thresholding powers. **(C)** Topological distribution of the constructed network, including the connectivity distribution and scale-free topology fitting. **(D)** Hierarchical clustering dendrogram of proteins and module assignment identified by dynamic tree cutting. Different colors indicate different co-expression modules. **(E)** Module eigengene adjacency heatmap showing the similarity and clustering relationships among identified protein modules.

SUPPLEMENTARY FIGURE 3 Network construction and module detection in metabolomic WGCNA. **(A)** Hierarchical clustering dendrogram of all samples used for metabolomic WGCNA. **(B)** Analysis of the soft-thresholding power for network construction. The left panel shows the scale-free topology fit index, and the right panel shows the mean connectivity under different soft-thresholding powers. **(C)** Topological distribution of the metabolomic network, including the connectivity distribution and scale-free topology fitting. **(D)** Hierarchical clustering dendrogram of metabolites and module assignment identified by dynamic tree cutting. Different colors indicate different co-abundance modules. **(E)** Module eigengene adjacency heatmap showing the relationships among metabolomic modules.

SUPPLEMENTARY FIGURE 4 Tissue-specific source tracing of WGCNA-defined proteomic modules. **(A)** Tissue-specific source tracing of proteins in WGCNA-defined proteomic modules. The distribution of tissue-specific proteins is shown for each module, with proteins further classified according to cellular topology, including secreted, membrane-associated, and membrane/secreted dual isoforms. The green module, which was clinically relevant to AMS-related phenotypes, is highlighted with a red box and was predominantly enriched in liver-derived secreted proteins.
