# Supplementary material for: Integrated proteomic and metabolomic analyses define the molecular architecture of acute mountain sickness
Source: Front Genet. 2026 Sep 1;17:1865110. doi: 10.3389/fgene.2026.1865110 (PMC13574784; doi:10.3389/fgene.2026.1865110)
Supplement: Supplementary file 1 [file Table1.docx]

**Supplementary table 1. Lake Louise Score symptom items and diagnostic definition of acute mountain sickness**

| **Item** | **Severity / classification** | **Score / definition** |
| --- | --- | --- |
| **Symptom scoring** | | |
| Headache | None | 0 |
| Headache | Mild headache | 1 |
| Headache | Moderate headache | 2 |
| Headache | Severe headache | 3 |
| Gastrointestinal symptoms | Good appetite / no nausea | 0 |
| Gastrointestinal symptoms | Poor appetite or mild nausea | 1 |
| Gastrointestinal symptoms | Moderate nausea or vomiting | 2 |
| Gastrointestinal symptoms | Severe nausea and vomiting, interfering with usual activities | 3 |
| Fatigue / weakness | None | 0 |
| Fatigue / weakness | Mild fatigue or weakness | 1 |
| Fatigue / weakness | Moderate fatigue or weakness | 2 |
| Fatigue / weakness | Severe fatigue or weakness, interfering with usual activities | 3 |
| Dizziness / lightheadedness | None | 0 |
| Dizziness / lightheadedness | Mild dizziness / lightheadedness | 1 |
| Dizziness / lightheadedness | Moderate dizziness / lightheadedness | 2 |
| Dizziness / lightheadedness | Severe dizziness / lightheadedness, interfering with usual activities | 3 |
| Sleep disturbance | Slept as well as usual | 0 |
| Sleep disturbance | Did not sleep as well as usual | 1 |
| Sleep disturbance | Woke many times and slept poorly | 2 |
| Sleep disturbance | Could not sleep at all | 3 |
| **Diagnostic definition** | | |
| non-AMS (nAMS) | Total score < 3 | Does not meet the diagnostic criteria for AMS |
| AMS | Total score ≥ 3, with headache present | Meets the diagnostic criteria for AMS |

Abbreviations: AMS, acute mountain sickness; nAMS, non-acute mountain sickness. Note: AMS was diagnosed according to the Lake Louise Score.
